# Pulse Wave Velocity: Methodology, Clinical Applications, and Interplay with Heart Rate Variability

**DOI:** 10.31083/j.rcm2507266

**Published:** 2024-07-17

**Authors:** Niklas Pilz, Viktor Heinz, Timon Ax, Leon Fesseler, Andreas Patzak, Tomas Lucca Bothe

**Affiliations:** ^1^Charité – Universitätsmedizin Berlin, Institute of Physiology, Center for Space Medicine and Extreme Environments Berlin, 10117 Berlin, Germany; ^2^Charité – Universitätsmedizin Berlin, Institute of Translational Physiology, 10117 Berlin, Germany; ^3^Department of Ophthalmology, Saarland University Medical Center, 66421 Homburg, Germany; ^4^School of Medicine, Western Sydney University, Sydney, NSW 2000, Australia

**Keywords:** pulse wave velocity, heart rate variability, measurement of pulse wave velocity, clinical applications of pulse wave velocity, interplay of pulse wave velocity and heart rate variability

## Abstract

Pulse wave velocity (PWV) has been established as a promising biomarker in cardiovascular diagnostics, providing deep insights into vascular health and cardiovascular risk. Defined as the velocity at which the mechanical wave propagates along the arterial wall, PWV represents a useful surrogate marker for arterial vessel stiffness. PWV has garnered clinical attention, particularly in monitoring patients suffering from vascular diseases such as hypertension and diabetes mellitus. Its utility extends to preventive cardiology, aiding in identifying and stratifying cardiovascular risk. Despite the development of various measurement techniques, direct or indirect tonometry, Doppler ultrasound, oscillometric analysis, and magnetic resonance imaging (MRI), methodological variability and lack of standardization lead to inconsistencies in PWV assessment. In addition, PWV can be estimated through surrogate parameters, such as pulse arrival or pulse transit times, although this heterogeneity limits standardization and, therefore, its clinical use. Furthermore, confounding factors, such as variations in sympathetic tone, strongly influence PWV readings, thereby necessitating careful control during assessments. The bidirectional relationship between heart rate variability (HRV) and PWV underscores the interplay between cardiac autonomic function and vascular health, suggesting that alterations in one could directly influence the other. Future research should prioritize the standardization and increase comparability of PWV measurement techniques and explore the complex physiological variables influencing PWV. Integrating multiple physiological parameters such as PWV and HRV into algorithms based on artificial intelligence holds immense promise for advancing personalized vascular health assessments and cardiovascular care.

## 1. Introduction

Recently, the use of pulse wave velocity (PWV) has transitioned from a predominantly research-focused tool to a marker of clinical importance [1, 2]. PWV has emerged as a key biomarker in cardiovascular diagnostics, offering critical insights into vascular health status [3]. Alterations in PWV indicate changes in arterial function, serving as a window into the cardiovascular system’s condition [3, 4, 5, 6, 7, 8].

PWV is the velocity at which the mechanical wave, generated by blood ejection from the heart, propagates along the arterial wall [9]. Additionally, PWV is intrinsically linked to the elasticity of the arterial vessels [10, 11, 12], whereby higher elasticity in younger, healthier vessels causes the pulse wave to travel more slowly. In comparison, increased arterial stiffness leads to faster pulse wave propagation, resulting in a higher PWV [13, 14]. Since the composition of arterial vessels is a crucial marker of cardiovascular health, PWV is an effective tool for evaluating cardiovascular risk and monitoring the progression of vascular diseases [2, 15, 16].

This review aims to highlight the importance of PWV as a cardiovascular marker, exploring its measurement techniques and current applications. Additionally, we will explore the interplay between heart rate variability (HRV) and PWV and its clinical importance. This review aims to provide a comprehensive overview that synthesizes existing knowledge while identifying goals for future research.

## 2. Approaches to Measuring Pulse Wave Velocity

Different measurement techniques have been developed to determine the PWV, which can be measured along various paths within the human body. The carotid–femoral PWV is measured between the carotid artery in the neck and the femoral artery in the groin. This path reflects the arterial stiffness of the central arteries, specifically the aorta, which is a key indicator of cardiovascular risk [17, 18]. In the brachial–ankle PWV measurement, the velocity is assessed between the brachial artery in the arm and the ankle arteries, thereby encompassing both the central and peripheral arterial paths [19]. Additionally, the cardio–ankle vascular index presents a unique approach by adjusting PWV for blood pressure variations, yielding a measure that reflects arterial stiffness from the heart to the ankle, independent of momentary blood pressure changes [20, 21].

The following section will explore these methods, their principles, advantages, and limitations (Table 1, Ref. [22, 23, 24, 25, 26, 27, 28, 29, 30, 31, 32, 33, 34, 35, 36, 37, 38, 39, 40, 41, 42, 43, 44, 45, 46, 47, 48, 49, 50, 51, 52, 53]).

**Table 1.** **Overview of different approaches to measure pulse wave velocity focusing on their principles, advantages, and possible disadvantages**.

Approach	Principle	Advantage	Disadvantages	Reference
Tonometry (direct)	Time-delay between two pressure sensors placed on the skin along an arterial vessel	Accurate and direct detection of PWV	Prone to artifacts; sensors must be placed correctly; affected by any movements	[22, 23, 24, 25, 26, 27]
Tonometry (indirect)	Time-delay between two light-sensors (photoplethysmography) on the skin along an arterial vessel	Accurate and indirect detection of PWV; less prone to artifacts	Indirect measurement of volume changes; sensors must be placed correctly; affected by stray light	[28, 29, 30]
Doppler ultrasound	Doppler effect	Real-time observation; depiction of specific arterial segment	Highly dependent on observer’s skills, transducer placement, and angle of wave entrance	[31, 32, 33, 34, 35, 36]
Oscillometer analysis	Fluctuations in pressure in the cuff during blood pressure measurement	Easy to perform; quick measurement	Potentially inaccurate, prone to movement artifacts	[37, 38, 39, 40, 41]
MRI	High-resolution images of the arterial walls	Observer independent; high reproducibility	Low accessibility; high costs	[42, 43, 44, 45, 46, 47, 48, 49]
PAT/PTT	PWV estimation using surrogate parameters	Easy to detect; possible use in wearable devices for long-term studies	Need for transfer functions, indirect and potentially inaccurate PWV measurements	[50, 51, 52, 53]

PWV, pulse wave velocity; MRI, magnetic resonance imaging; PAT, pulse arrival time; PTT, pulse transit time.

### 2.1 Tonometry (Direct)

This approach is recognized as the most accurate method for determining PWV. This technique measures the time required for the pulse wave to traverse from one arterial site to another. Pressure sensors placed directly on the skin above two arteries detect volume changes on the vessel propagated through the tissue. The time delay between the two sensors directly represents the PWV [22, 27, 54]. Commonly, the time difference between the carotid and femoral arteries is observed [23].

The technique requires a high level of observer skill and experience to be performed correctly. The accuracy of tonometry is highly dependent on the proper placement of the sensor and the quality of the arterial signal obtained. Factors such as patient movement and external pressure on the artery strongly affect the measurement [25, 26]. Despite these challenges, direct tonometry remains the gold standard in PWV measurement [24, 27].

### 2.2 Tonometry (Indirect)

Indirect tonometry is a derivative measurement of direct tonometry. Instead of pressure sensors placed on the skin, indirect tonometry utilizes pulse-plethysmography (light) sensors to detect volume changes along an artery. This is performed assuming that any change in intra-arterial pressure coincides with a simultaneous shift in arterial cross-section and, therefore, its reflectance. These sensors can be placed on any artery reachable by visual light, such as the carotid or radial artery in the index finger [28, 29].

The approach requires less skill from the observer but relies on the correct and consistent placement of the sensors. However, stray light and darker skin can impede measurement quality or even prohibit measurement, limiting the approach. Still, technical solutions can be found to allow light-based PWV detection in people of all skin shades [30].

### 2.3 Doppler Ultrasound

This technique leverages the Doppler effect, which refers to the change in wavelength for moving wave sources (e.g., pulse wave) relative to the observer [32]. This physical effect can be leveraged to detect flow changes within the vessel in the ultrasound, indicating the arrival of the pulse wave. Therefore, it is possible to detect the PWV between two arterial points found in the ultrasound. One of the primary advantages of Doppler ultrasound is its capacity to deliver real-time, detailed insights into blood flow dynamics, including flow velocity and turbulence. It also enables the assessment of PWV across specific arterial segments, aiding in the accurate localization of vascular disease and facilitating the development of personalized therapeutic plans [31, 33, 34, 36, 55].

However, the accuracy of Doppler ultrasound measurements can be affected by various factors, such as the angle at which the ultrasound wave enters the body, the operator’s expertise, and the patient’s body composition [32, 35, 56, 57].

### 2.4 Oscillometric Method

This technique employs cuffs, similar to those used in blood pressure measurements, which are placed around the patient’s arms and/or legs [24, 37, 38, 39, 40]. These cuffs detect small fluctuations in the cuff pressure caused by the blood’s pulse waves when inflated to sub-diastolic pressures [41, 58]. Subsequently, PWV is calculated by analyzing the time delay between these fluctuations at various positions.

A key aspect of the oscillometric method is its simplicity and speed, making it ideal for clinical office examinations. This expediency is of particular value in high-throughput clinical environments where swift screening can facilitate early detection of cardiovascular risks in a preventative setting. Furthermore, this method uses automated devices, which reduces operator-dependent variability [37, 39, 40].

Despite its utility and patient-friendly nature, the oscillometric method is viewed as less accurate than more sophisticated techniques [24, 59]. Factors including artifacts from the measurement process, the patient’s body position, and the correct sizing and placement of the cuff can all affect the precision of the oscillometry-derived PWV readings [24, 59, 60].

### 2.5 Magnetic Resonance Imaging

Magnetic resonance imaging (MRI) measures PWV and detailed anatomical and functional data, allowing for a comprehensive assessment of vascular health. MRI determines the PWV by capturing high-resolution images of the arterial walls and blood flow [42, 43].

The technique involves using phase-contrast MRI, which visualizes and quantifies the blood flow speed through the arteries. This enables the PWV to be calculated from the time shift between different waveforms over a specific segment of the arterial system [42]. Thus, assessing PWV directly within central vessels represents an advantage over other methods [44, 45, 46]. Further, MRI can provide accurate and reproducible measurements of arterial distension and blood flow without the influence of external factors such as operator skill or sensor placement [47, 48, 49].

MRI systems are constrained by their high costs and the need for specialized infrastructure and trained staff, limiting their availability to well-equipped clinical centers and research institutions. Additionally, MRI examinations are more time-consuming than other PWV measurement techniques [61, 62, 63].

### 2.6 Estimating Pulse Wave Velocity Using Surrogate Parameters

In addition to direct measurement techniques, PWV can be estimated using surrogate parameters such as pulse arrival and transit times. These indirect approaches have gained popularity due to their simplicity and the minimal equipment required. Estimating the PWV provides an inferred measure of arterial health, offering an alternative when a direct PWV measurement is unavailable [50, 51, 64].

Pulse arrival time refers to the time it takes for the pulse wave to travel from the heart to a peripheral site. Typically, it is detected between the Q- or R-wave in an electrocardiogram (ECG) and the onset of the pulse wave captured at a peripheral site (e.g., finger) using a photoplethysmography sensor. This measurement reflects the combined effects of cardiac ejection and arterial stiffness, making it a useful proxy for vascular property changes, particularly in longitudinal studies with established individual baselines [50, 52, 53, 65].

On the other hand, pulse transit time describes the time it takes for the pulse wave to travel between two sites in the arterial system. The pulse transit time excludes the intracardial component, which is part of the pulse arrival time. Typically, it is calculated as the interval between blood ejection from the heart and the arrival of the pulse wave at a distal site [64, 66].

**Fig. 1.**
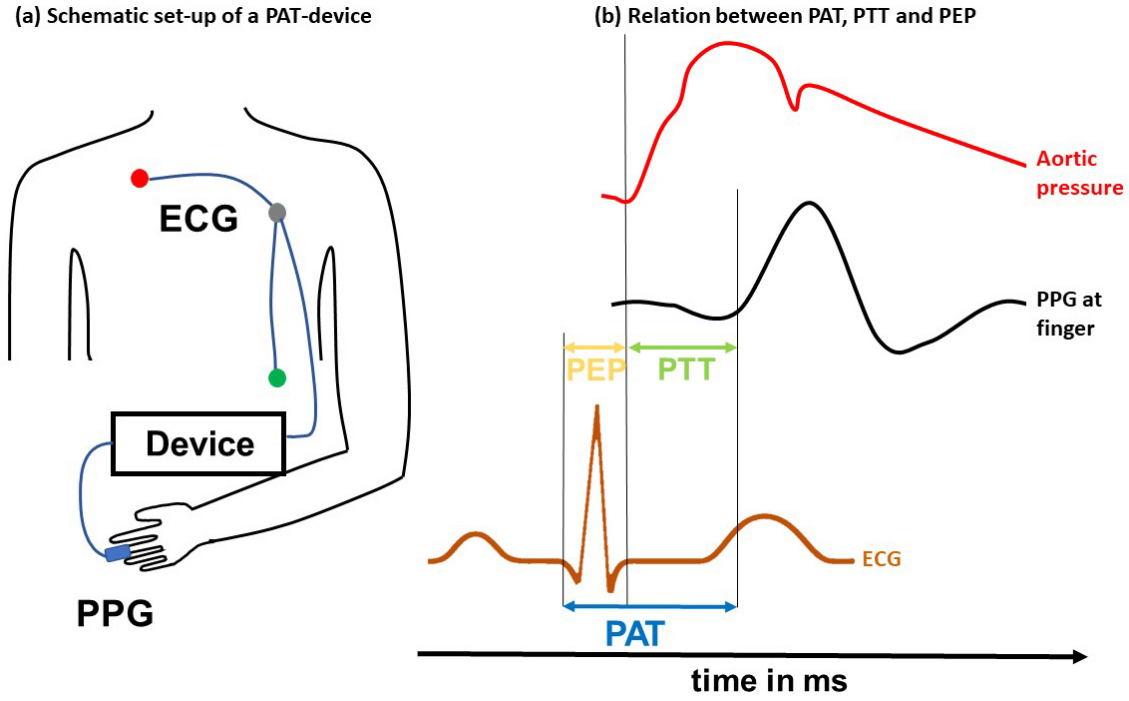
**PWV surrogate parameters.** The left panel (a) shows the schematic set-up of a pulse arrival time measurement device. An electrocardiogram (ECG) is placed on the patient’s thorax. A photoplethysmography (PPG) sensor is placed at the peripheral side (e.g., at the finger). Both devices are interconnected with a data recorder. The right panel (b) shows the relationship between the pre-ejection period, pulse transit time, and pulse arrival time. The pulse arrival time is typically detected between the Q- or R-wave in an ECG and the onset of the pulse wave at the peripheral site captured, e.g., using a photoplethysmography sensor. The pulse transit time is measured between the sites (heart and finger) in the arterial system. The pre-ejection period is the delay between the Q- or R-wave and the start of blood ejection into the aorta. Modified from Pilz *et al*., 2022 [50]. PEP, pre-ejection period; PTT, pulse transit time; PAT, pulse arrival time; PWV, pulse wave velocity.

The interval between the Q- or R-wave and blood ejection from the heart differentiates pulse arrival time from pulse transit time and is known as the pre-ejection period [67] (Fig. 1, Ref. [50]).

Both pulse arrival time and pulse transit time offer a noninvasive estimation of PWV, with the pulse transit time being more closely related to the true PWV, although it is methodologically more difficult to obtain [67]. Pulse arrival and transit times use transfer functions to estimate the distance between the heart and the peripheral sensor [52, 53]. These approaches are particularly advantageous for large-scale epidemiological studies and for use in wearable technology, where ease of use and patient comfort are essential [50, 65, 68].

## 3. Clinical Applications of Pulse Wave Velocity

PWV has emerged as a valuable clinical medicine tool, particularly in cardiovascular health. Its primary application lies in its ability to provide a noninvasive assessment of vessel status as it reflects arterial stiffness [3, 11, 14, 69, 70, 71].

Artery stiffening is a marker of vascular aging and an independent predictor of cardiovascular events and all-cause mortality. Thus, by quantifying arterial stiffness, PWV provides clinicians with actionable data that can contribute to the decision-making process regarding initiating or intensifying treatment regimens, particularly in managing hypertension and other cardiovascular risk factors [71, 72, 73, 74, 75].

Moreover, longitudinal studies have demonstrated that changes in PWV over time can indicate the progression or regression of hypertensive vascular disease, underscoring its potential in patient follow-up and disease management [76, 77, 78, 79, 80].

Further, PWV assessments might capture the early vascular changes that precede atrial fibrillation, thus allowing the early diagnosis of potential at-risk patients through multiple measurements. However, further research is required to observe the relationship between arterial stiffness and atrial fibrillation [72].

Additionally, PWV measurement is instrumental in managing patients with diabetes mellitus, where accelerated arterial stiffening is a common complication, aiding in the early detection and treatment of cardiovascular issues [81, 82, 83, 84, 85, 86].

In patients with chronic kidney or end-stage renal disease, PWV assessment helps evaluate the increased cardiovascular risk associated with these conditions, guiding the management and therapeutic approach [75, 87]. The ability of PWV to respond to therapeutic interventions also offers a quantifiable endpoint in clinical trials assessing the efficacy of novel cardiovascular drugs or interventions [6, 10]. However, no large-scale, controlled intervention study has yet shown the efficacy of PWV as a primary treatment target for cardiovascular risk reduction.

The application of PWV extends to cuff-less continuous blood pressure monitoring, which serves as a surrogate parameter for arterial blood pressure. This innovative approach leverages the correlation between arterial stiffness and blood pressure levels. Hence, continuously measuring PWV makes it possible to estimate blood pressure changes noninvasively, cuff-less, and constantly (beat-to-beat) [50, 65, 88, 89]. However, it is important to note that the European Society of Hypertension does not currently recommend clinical decision-making solely based on blood pressure values obtained from cuff-less blood pressure measurements [90, 91].

## 4. Challenges and Limitations of Pulse Wave Velocity

A major challenge in measuring PWV is the methodological variability across different techniques, which can produce disparaging results. This variability introduces potential inconsistencies in data capture and interpretation, complicating the comparison of PWV values between studies and clinical practice. The wide range of measurement approaches and procedures highlights the complexity of achieving consistent and comparable PWV assessments. To address this challenge, it is crucial to clearly report the measurement method used in both scientific publications and clinical report letters; develop conversion tables to facilitate value comparison across different measurement approaches; pursue consensus on standardizing approaches while acknowledging that each method has its own set of advantages and disadvantages [27, 92, 93, 94]. While an ideal method may not exist due to constraints such as cost and reliability, adopting a flexible approach that tailors the use of different techniques to specific needs—ranging from screening large patient populations to addressing particular questions in a smaller subset of patients and differentiating between routine clinical care and research—may be more practical.

Another limitation in PWV assessment arises from the influence of various factors on its readings. Variables, including current blood pressure, heart rate, and vascular tone, can strongly affect PWV. Acute fluctuations in blood pressure can cause immediate, although reversible, changes in arterial stiffness, highlighting the need for meticulous consideration of these factors during the assessment and interpretation of PWV data [53, 95, 96, 97, 98, 99]. Establishing consistent measurement conditions may offer a viable approach to mitigate the effects of these variables on PWV measurements.

Variability in PWV measurements can also stem from anatomical differences in arterial pathways and the methodologies used for distance calculations [100, 101]. The carotid–femoral segment, commonly assessed for PWV, might not accurately represent the stiffness of peripheral arteries, which holds clinical importance for specific groups such as individuals with diabetic microangiopathy or peripheral arterial occlusive disease [101, 102, 103]. Moreover, discrepancies in distance measurements between pulse recording sites can introduce errors in PWV calculations, thereby affecting their clinical applicability [27, 104]. Advances in imaging technology and the standardized use of body surface landmarks could enable more precise localization and evaluation of arterial segments, potentially minimizing variability due to anatomical differences [102, 104, 105, 106].

Patient-related factors, such as obesity, also pose challenges [107, 108]. These conditions may impede accurate sensor placement or signal acquisition, particularly in methods such as tonometry, thereby affecting the reliability of PWV measurements [27, 109].

In summary, its measurement has various sources of PWV variability and methodological complications. Beyond that, only very few devices are properly validated according to the latest guidelines [110]. These reasons lead to a limited reproducibility of PWV measurements and might confound individual risk signals within the noise of uncontrolled variability [111]. This could be why no randomized, large-scale intervention study, primarily aiming at the PWV as a treatment target, has yet to show a reduction in cardiovascular risk or improved overall mortality.

## 5. Combination of Heart Rate Variability and Pulse Wave Velocity

As both are vital cardiovascular health and autonomic function indicators, the combination of HRV and PWV has garnered interest in cardiovascular research [96, 112, 113].

HRV, which measures the variability in heartbeat intervals, reflects the autonomic nervous system’s control over cardiac rhythm [114, 115, 116]. It plays a central role in the modulation and alteration of cardiac deformation and contractility, as evidenced by echocardiographic techniques. Furthermore, HRV is instrumental at the adaptive cardiac chamber activation level in response to autonomic tone dysfunction and might even anticipate pro-arrhythmic atrial effects and a higher incidence of syncope recurrence in the overall population, particularly in diabetic patients [117, 118, 119]. These findings underscore the importance of HRV in reflecting autonomic nervous control and its predictive value regarding cardiac events and dysfunction in adults.

Research suggests that there might be a complex and bidirectional interplay between HRV and PWV. Increased arterial stiffness, as indicated by higher PWV, can lead to changes in blood pressure dynamics. Subsequently, it may influence cardiac autonomic control, thereby affecting HRV. Lower HRV, indicative of an altered autonomic function caused by conditions such as atherosclerosis in the aorta, has been associated with higher PWV in various population studies [120, 121, 122].

The autonomic nervous system is central in regulating vascular tone and heart rate. As reflected by changes in HRV, alterations in autonomic balance influence arterial stiffness, either through direct effects on the vascular smooth muscle cells or indirectly via heart rate and blood pressure changes [113, 123].

The relationship between HRV and PWV extends beyond their roles as health markers, shedding light on the interconnectedness of cardiac and vascular functions and their collective representation of overall cardiovascular risk. However, it is important to acknowledge that the HRV–PWV relationship might be influenced by numerous confounders, including age, diabetes mellitus, blood pressure, physical fitness, existing pathologies, and natural interpersonal variation [96, 112, 113, 124]. Studies suggest that the autonomic nervous system does influence pressure-independent aortic stiffness in young, healthy subjects [124]. Therefore, while the HRV–PWV association offers valuable insights, its interpretation must be contextualized within the unique health profiles and risk factors of individual patients, which emphasizes the need for further research into this relationship to offer a nuanced understanding of its clinical assessment [120, 125, 126].

## 6. Future Directions in Pulse Wave Velocity Research

A primary area of focus should be standardization and increased comparability between different assessments of PWV measurement techniques. Given the various methods currently used, establishing uniform protocols and guidelines and using validated devices is critical for enhancing the comparability of PWV data across diverse studies and clinical scenarios [27, 92, 93, 110].

Moreover, there is a pressing need for further research to illuminate the physiological factors that influence PWV. This encompasses examining the effects of demographic, genetic, and lifestyle variables [97]. Conducting longitudinal studies that track PWV changes over time, and their correlation with cardiovascular events and outcomes in varied populations will shed light on the predictive value of PWV [127].

Clinical trials are also essential to assess the effectiveness of interventions designed to mitigate arterial stiffness, as quantified by PWV. These interventions might range from pharmacological treatments and lifestyle alterations to innovative therapeutic strategies. Thus, understanding how these interventions impact PWV could facilitate the creation of personalized treatment plans and enhance the management of patients at increased cardiovascular risk.

However, integrating PWV assessment into routine clinical practice warrants additional exploration. This includes identifying the most efficacious, user-friendly, and pragmatically feasible methods for incorporating PWV measurements into prevailing cardiovascular risk models and clinical procedures, thereby maximizing the utility of PWV in patient care.

Furthermore, technological advancements and machine learning hold promise for developing advanced tools for PWV measurement and analysis. These innovations could enable more precise, noninvasive, and user-friendly methods for evaluating arterial stiffness, both in clinical environments and at home. Integrating diverse parameters such as HRV into sophisticated models powered by artificial intelligence may offer deeper insights into cardiovascular health and individual risk profiles, paving the way for more nuanced and effective patient care strategies [128, 129].

## 7. Summary and Conclusions

PWV represents an important biomarker for arterial stiffness, potentially enhancing cardiovascular diagnostics and providing further insights into vascular health. Despite the availability of various measurement techniques, the field faces challenges due to the need for more standardization and comparability, validation of devices, and the inherent limitations of each method. This results in a large variability and poor reproducibility of PWV assessments. The emergence of new technologies and the application of artificial intelligence are poised to create innovative tools that utilize comprehensive parameters, including HRV and PWV, for a holistic assessment of cardiovascular health. Specifically, pinpointing PWV in distinct arterial segments could be particularly beneficial for individuals with conditions such as diabetes-induced vessel disease, offering tailored diagnostic insights.

Acknowledging the predictive value of PWV in predicting cardiovascular events and mortality, this review emphasizes PWV’s marked potential for modern cardiovascular risk stratification and management. Leveraging PWV could refine cardiovascular prevention and treatment strategies, improving patient outcomes through targeted and sophisticated approaches. Further integration of reliable and precise PWV determination in everyday clinical practice holds promise for advancing the management of cardiovascular diseases and enhancing patient care.
